# Cartilage thickness can be accurately measured intraoperatively in total knee arthroplasty: A step further in calipered kinematic alignment

**DOI:** 10.1002/jeo2.70155

**Published:** 2025-01-26

**Authors:** Giancarlo Giurazza, Stefano Campi, Michael T. Hirschmann, Edoardo Franceschetti, Andrea Tanzilli, Pietro Gregori, Michele Paciotti, Biagio Zampogna, Rocco Papalia

**Affiliations:** ^1^ Department of Orthopaedic and Trauma Surgery Fondazione Policlinico Universitario Campus Bio‐Medico Roma Italy; ^2^ Department of Medicine and Surgery Research Unit of Orthopaedic and Trauma Surgery, Università Campus Bio‐Medico di Roma Roma Italy; ^3^ Department of Orthopaedic Surgery and Traumatology Kantonsspital Baselland (Bruderholz, Liestal, Laufen) Bruderholz Switzerland; ^4^ Department of Medicine and Surgery University of Basel Basel Switzerland

**Keywords:** cartilage thickness measurement, electrocautery tip method, intraoperative techniques, kinematic alignment (KA), total knee arthroplasty (TKA)

## Abstract

**Introduction:**

Kinematic alignment (KA) in total knee arthroplasty (TKA) is by definition a pure femoral resurfacing procedure aiming to restore the individual prearthritic anatomy. However, when a 2 mm compensation is systematically used on the worn side, the variability in cartilage thickness in the unworn compartment might alter the accuracy of the technique. This study aimed to validate two intraoperative femoral cartilage thickness measurement techniques by comparing them to the photographic method, which measures cartilage thickness through pixel analysis of bone‐cut images. The study hypothesized that the two intraoperative methods are comparable and similarly accurate within 0.5 mm of the photographic method.

**Methods:**

Seventy cartilage thickness measurements from seventy patients with end‐stage knee osteoarthritis were prospectively collected. Two intraoperative techniques were evaluated: the electrocautery tip method (Method A) and the ruler method (Method B), performed before and after distal femoral bone resections, respectively. The postoperative photographic analysis (Method C) served as the reference method. Measurements were rounded to the nearest 0.5 mm for consistency. Data were analyzed using Kruskal–Wallis test, Wilcoxon rank‐sum tests, Spearman's rank correlation, percentage of agreement and intraclass correlation coefficients (ICCs).

**Results:**

No significant differences were observed between Method A and Method B in measuring femoral cartilage thickness. Agreement with Method C was 100% for Method B and 85% for Method A. In the 15% of discordant cases, Method A overestimated the measurements by one category of 0.5 mm compared to Method C. Correlation coefficients between the methods were high (*ρ* = 0.88−1.0). Intra‐ and interobserver reliability was high for all methods (ICCs 0.91–0.95).

**Discussion:**

Both intraoperative methods are reliable and comparable to the photographic method when rounded to the closest 0.5 mm, with no significant differences among them. The electrocautery method has the added advantage of measuring cartilage thickness before bone cuts are performed.

**Level of Evidence:**

Level IV.

**Abbreviations**
ICC: intraclass correlation coefficients
IQR: interquartile range
KA: kinematic alignment
MRI: magnetic resonance imaging
SD: standard deviation
TKA: total knee arthroplasty

## INTRODUCTION

The number of total knee arthroplasties (TKAs) has risen steadily in recent years, reflecting advancements in surgical techniques and increased confidence in safety and outcomes [15]. Despite mechanical alignment having long been regarded as the gold standard [23], it is associated with a dissatisfaction rate of up to 20% [7, 40, 41], especially among patients with varus phenotypes [14], which may benefit the most of more personalized surgical approaches [13].

Kinematic alignment (KA) has recently gained attention as a viable alternative [9, 10, 12, 30, 36, 46, 49], offering benefits such as improved pain relief, range of motion and a more natural knee sensation [2, 5, 6, 8, 38, 47]. KA aims to replicate prearthritic tibiofemoral alignment and joint laxity [32, 45] using a true femoral resurfacing technique, assuming a consistent cartilage thickness of 2 mm for the distal and posterior condyles [39]. However, interindividual variability in cartilage thickness may affect the accuracy of KA [17], making intraoperative evaluations of cartilage thickness essential to truly express the KA philosophy at its full potential.

Several methods, including ultrasounds, stereomicroscopy and the needle probe technique, have been described to measure cartilage thickness [16, 24, 25, 27], though their time‐consuming nature and the need for external equipment makes them impractical in an operating room setting. A cartilage thickness gauge has been included in their KA instrumentation by Evolution® **(**MicroPort Orthopaedics Inc.), although, to the best of our knowledge, data on its accuracy has never been reported.

This study aims to evaluate and validate two universally applicable intraoperative methods for measuring femoral cartilage thickness, by comparing them with the postoperative photographic analysis [48], considered as the most accurate method. The study hypothesized that the two intraoperative cartilage measurement methods are comparable and highly accurate in determining cartilage thickness within 0.5 mm of the photographic method.

## MATERIALS AND METHODS

### Study design and participants

Institutional review board approval (IRB No. PAR 24.22 OSS) was granted for this study and written consent was obtained from all participants. No financial incentives were provided for participation. Data were prospectively collected from patients diagnosed with end‐stage knee osteoarthritis (Kellgren–Lawrence grade IV) undergoing TKA at our Institution (Fondazione Policlinico Universitario Campus Bio‐Medico) between April and May 2024. Measurements were conducted on the distal femoral condyle of the unworn knee compartment. Patients with tricompartmental knee osteoarthritis or chondral defects in weight‐bearing areas of the unworn compartment were excluded from the study. About 70 patients met the aforementioned criteria.

### Measurement techniques

**Figure 1:**
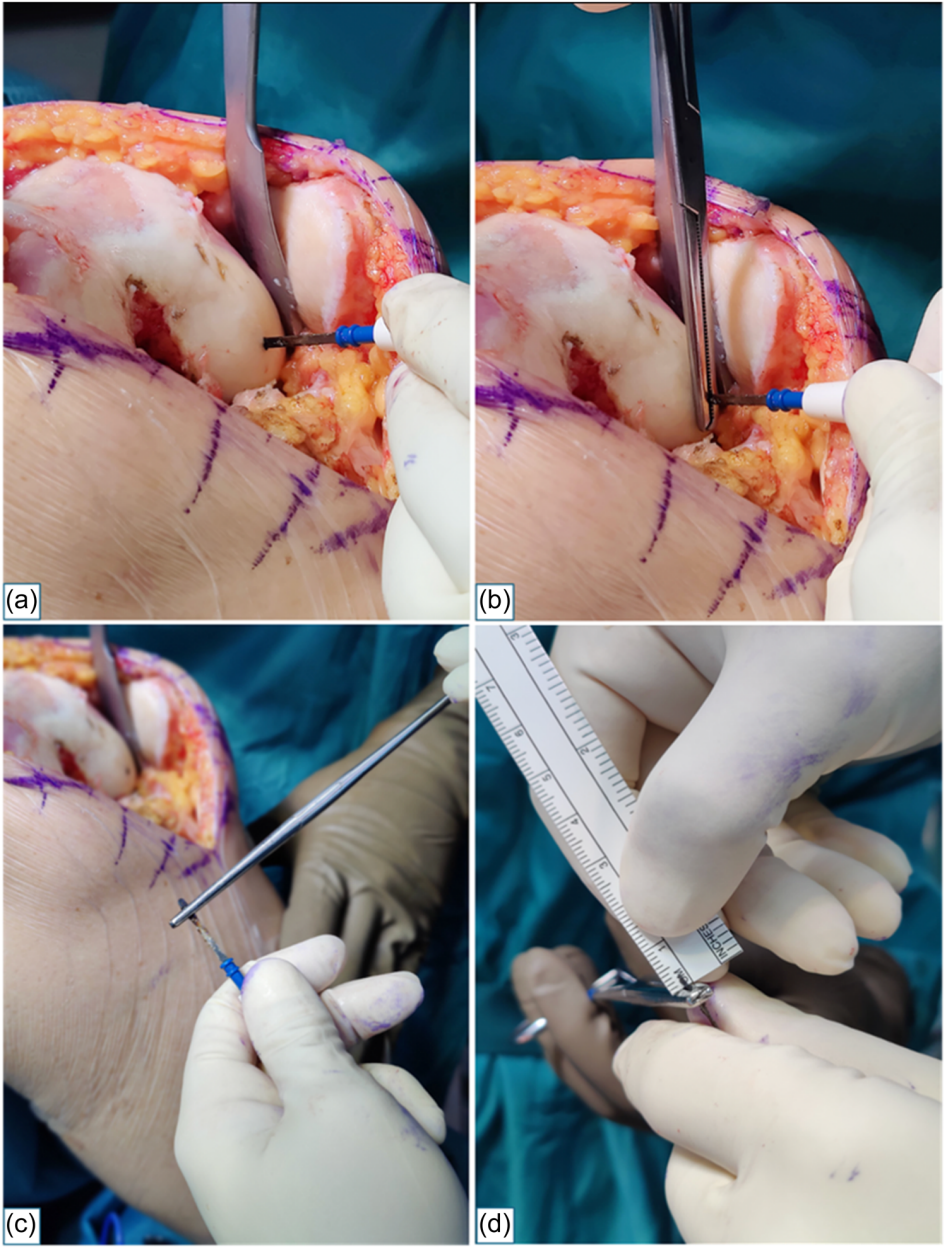
Method A. The tip of the electrocautery is dipped through cartilage, until contact with the underlying subchondral bone (a). The electrocautery tip is clamped with a Kocher forceps and withdrawn (b and c). The portion of the electrocautery tip extending beyond the Kocher forceps is measured with a millimetre ruler (d).

**Figure 2:**
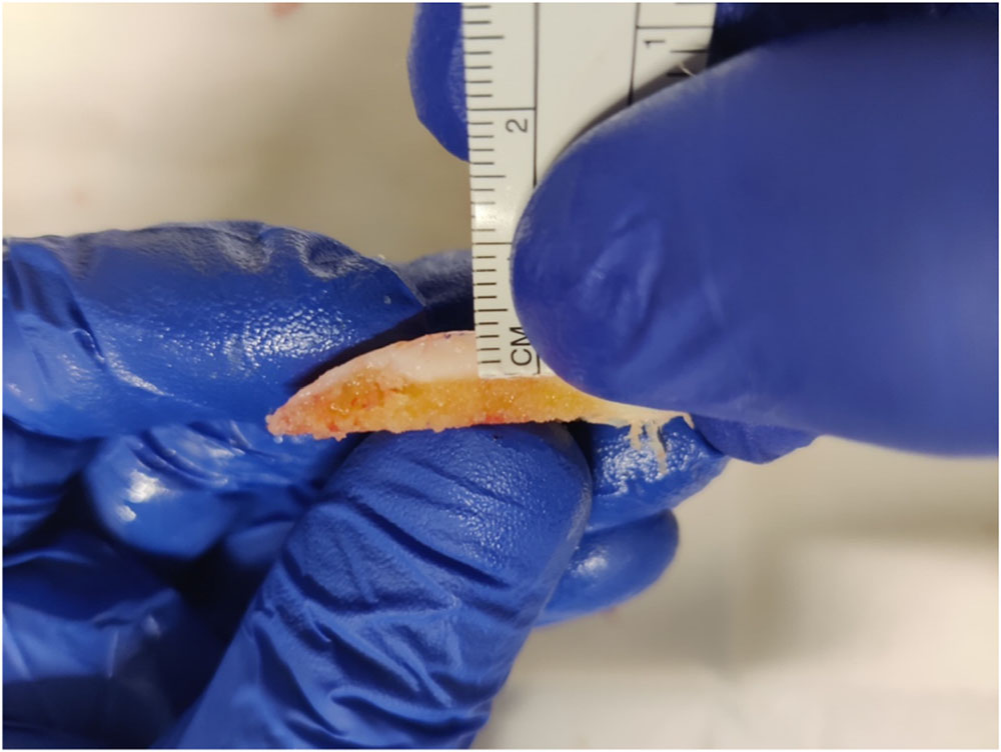
Method B: The bone–cartilage biscuit is halved perpendicular to the cut surface. Cartilage thickness is measured at the bone–cartilage interface with a millimetre ruler.

All intraoperative measurements were performed twice by a single surgeon (E. F.) and then repeated by an additional surgeon (G. G.) using two distinct methods:

‐ **Method A:** Before performing the bone cuts, the most distal point of the unworn distal femoral condyle was identified with the knee flexed at 90° as the first contact point of an osteotome advanced in an anteroposterior direction, perpendicular to the tibial surface. Subsequently, the tip of the electrocautery was dipped into cartilage at the identified most prominent point, advancing until it made contact with the underlying bone (Figure 1a). The electrocautery tip was then clamped with a Kocher forceps, firmly pressed on the cartilage surface and withdrawn (Figure 1b,c). The portion of the electrocautery tip extending beyond the Kocher forceps was then measured with a millimetre ruler (Figure 1d).
‐ **Method B:** Following the bone cuts, the bone–cartilage ‘biscuit’ was carefully divided into two symmetrical halves by first incising the cartilage perpendicularly to the cut surface at its most prominent point using a scalpel, then cutting through the bone with a thin osteotome. A millimetre ruler was then aligned with the cartilage to measure the distance from its most prominent point to the bone–cartilage (Figure 2) and a photograph was taken with a 100‐megapixel digital camera at a right angle to the cartilage surface.

**Figure 3:**
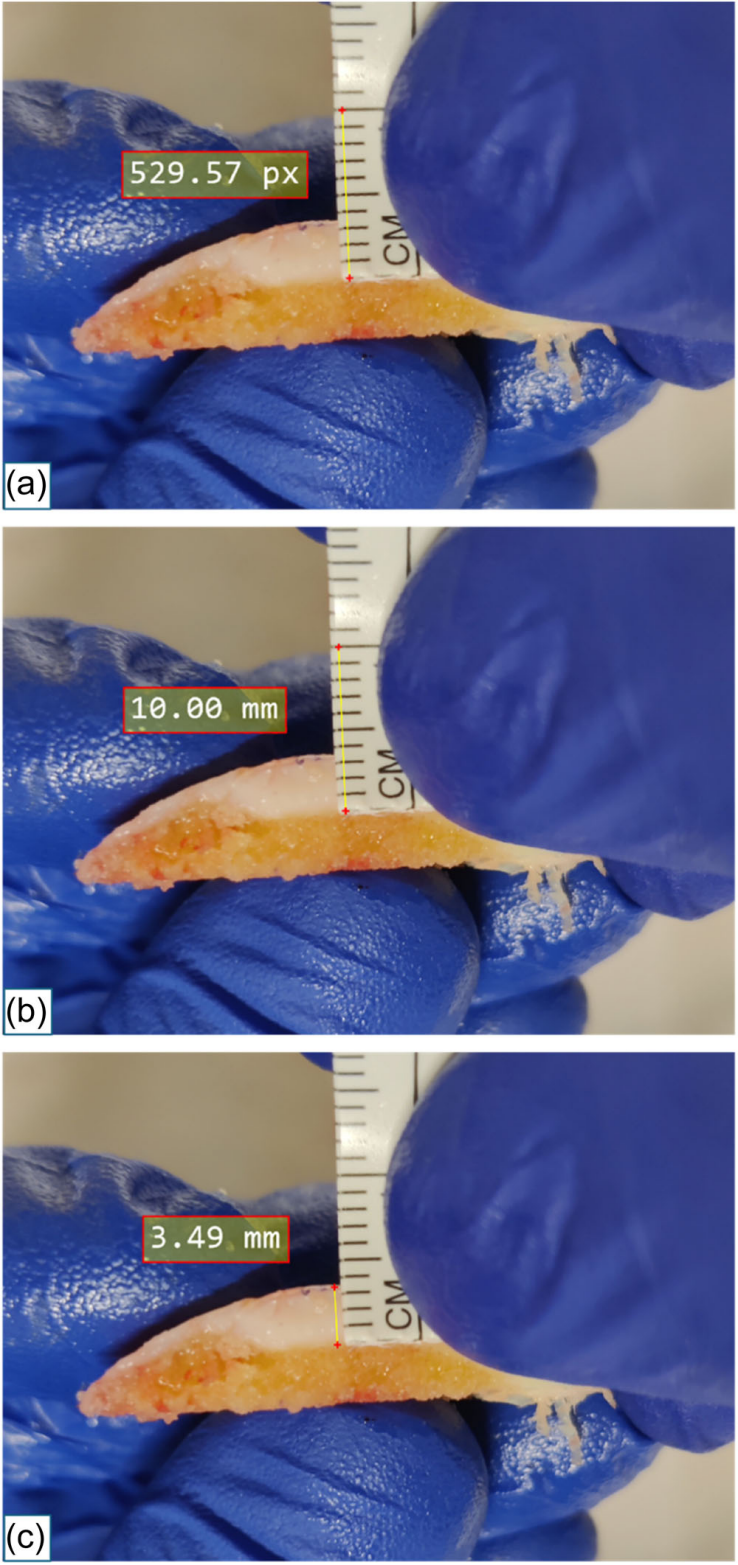
Method C: Intraoperative images are uploaded to a PACS system and the measuring tool is calibrated using the millimetre ruler (a and b). Cartilage thickness is measured at the most prominent point at the bone–cartilage interface (c). PACS, picture archiving and communication system.

After surgery, the images were uploaded to a picture archiving and communication system and cartilage thickness was measured as the distance from its most prominent point to the bone–cartilage interface (Figure 3), using the millimetre ruler to calibrate the measurement tools (**Method C**). Measurements with Method C were performed twice by the senior author (E. F.), with a 6‐week interval. Additionally, a second surgeon (G. G.) performed the same measurements to assess interobserver reliability.

Given that both intraoperative techniques rely on the use of a ruler with 1‐millimetre increments, and considering a ‘tolerated error’ in conventional KA TKA technique of 0.5 mm [21, 44], all photographic measurements were rounded to the nearest 0.5 mm for consistency.

### Data analyses

Descriptive statistics, including mean, median, range, standard deviation and interquartile range, were calculated for all variables. All data analyses were conducted using STATA 18 Software (StataCorp LLC). A Kruskal–Wallis test was performed to assess differences in cartilage thickness measurements among the three methods. To evaluate pairwise comparisons between the methods, Wilcoxon rank‐sum tests were employed. Additionally, Spearman's rank correlation coefficient was used to quantify the concordance between measurements obtained with Method A and Method B, Method A and Method C and Method B and Method C. The significance level for all tests was set at *p* < 0.05.

An a priori power analysis, conducted using G*Power software, indicated that a sample size of 64 patients per group was required to achieve 80% power, assuming an effect size of 0.5 and an α‐error of 0.05.

Both intra‐ and interobserver reliability of the three measurement methods were assessed by calculating intraclass correlation coefficients (ICCs).

## RESULTS

Patients' demographic characteristics, including age, height, weight and body mass index, are summarized in Table 1.

**Table 1:** Patient demographics (mean, SD).

	Total (*n* = 70)	Men (*n* = 39)	Women (*n* = 31)	*p* value men vs. women
Age (years)	69.5 (7.7)	70.7 (6.8)	68.4 (7.9)	0.331
Height (cm)	169.2 (10.2)	172.2 (8.1)	167.1 (7.3)	
Weight (kg)	81.5 (17.4)	83.5 (15.4)	78.6 (14.4)	
BMI	27.9 (6.2)	28.2 (5.2)	27.2 (4.7)	

Abbreviations: BMI, body mass index; SD, standard deviation.

The median cartilage thickness of the distal femoral condyle, as measured by each method and rounded to the nearest 0.5 mm, is shown in Table 2.

**Table 2:** Median values of cartilage thickness using methods A, B and C with pairwise comparison (Wilcoxon rank‐sum test) and overall comparison (Kruskal–Wallis test).

Method	Median (Q1–Q3)	*p* value A vs. B	*p* value A vs. C	*p* value B vs. C	*p* value A vs. B vs. C
Method A	2.5 (2–3)	0.397			
Method B	2.5 (2–3)		0.397	1.000	0.530
Method C	2.5 (2–3)				

Pairwise comparisons using Wilcoxon rank‐sum tests showed no significant differences between Method A and Method B (*p* = 0.397), Method A and Method C (*p* = 0.397) or Method B and Method C (*p* = 1.000). No statistically significant differences were found among Method A, B and C in the overall comparison, as determined by the Kruskal–Wallis test (*p* = 0.530).

Spearman correlation analysis showed very strong [18] positive correlations between Method A and Method B (*ρ *= 0.940, *p* < 0.0001) and between Method A and Method C (*ρ *= 0.940, *p* < 0.0001). Method B and Method C were perfectly correlated (*ρ *= 1.000, *p* < 0.0001), indicating complete agreement between these two methods. Percentage agreement analysis further confirmed these findings, with 100% agreement between Methods B and C and 85% agreement between Method A and the other two methods. In the 15% of discordant cases, Method A overestimated cartilage thickness by 0.5 mm compared to Methods B and C.

Intra‐ and interobserver reliability were excellent for all methods, with ICCs of 0.93 and 0.92 for Method A, 0.94 and 0.91 for Method B and 0.95 and 0.93 for Method C, respectively.

## DISCUSSION

The main result of the current study was that both intraoperative measurement methods were accurate in determining cartilage thickness within 0.5 mm of the photographic method. In addition, no statistically significant differences were found between the two intraoperative measurement methods.

A major limitation of previously described cartilage measurement techniques [16, 24, 25, 27] was their impracticality during surgery, primarily due to the time required and the need for specialized equipment. In contrast, the two intraoperative methods described in this study effectively address these challenges. Furthermore, the electrocautery technique (Method A) offers the additional advantage of measuring cartilage thickness in the unworn compartment before bone cuts are made, thereby avoiding the need for potential bone recuts and compensatory adjustments. Three key factors may explain the overestimation of cartilage thickness observed in 15% of the measurements with Method A compared to Method B (*p* = 0.188). First, there may be partial penetration of the subchondral bone by the tip of the electrocautery, which can occur to some extent in osteoporotic patients. Second, there could be a gap between the cartilage surface and the Kocher clamp. Finally, the cartilage layer might not be penetrated perpendicularly to the cut surface, resulting in an oblique measurement. However, even when overestimation occurred, it was limited to one 0.5 mm category, making the method sufficiently accurate for estimating cartilage thickness for surgical purposes [21, 44], as it falls within the tolerance error of the KA technique, and its integration may help prevent errors of much larger magnitude. This method, conceptually similar to the cartilage thickness gauge proposed by MicroPort Orthopaedics, offers the added benefits of being validated and universally applicable, without depending on any specific company's instrumentation. Additionally, using the rounded tip of the electrocautery, rather than the pointed tip of the cartilage gauge, helps reduce the risk of partially penetrating the subchondral bone, which could otherwise lead to a greater overestimation of cartilage thickness.

Giurazza et al. [17] have shown significant variability in femoral cartilage thickness among individuals, with mean values reported as high as 4.4 ± 1.4 mm [28]. This finding challenges one of the core principles of KA, which assumes a consistent 2 mm compensation for cartilage wear. According to basic trigonometric concepts, the entity of the angular change in Lateral Distal Femoral Angle, femoral component rotation and trochlear groove orientation [19, 22, 42] is simply calculated as the arctangent of the ratio between the error in cartilage thickness compensation and the intercondylar distance (*α *= tan^−^^1^ [error in cartilage compensation/intercondylar distance]). Consequently, a given error in cartilage thickness compensation will have greater consequences in narrow femurs, frequently encountered in the female population [29]. This may help explain the findings of Nam et al. [37], where posteromedial cartilage wear had a greater effect on femoral component rotation in females compared to males in the varus population.

Integrating straightforward and practical intraoperative cartilage measurement methods into routine practice may allow for more accurate and effective joint reconstruction, enabling better accommodation of individual anatomical differences, reducing errors that could affect the femoral joint line [31], prosthetic component rotation [35] and patellar tracking [1, 4, 34] and potentially leading to improved clinical outcomes.

After accurately measuring cartilage thickness in the unworn compartment of the knee, the key question arising is whether we can really assume that the cartilage in the worn compartment was originally the same thickness before the onset of osteoarthritis. Furthermore, current KA instrumentation offers only a 2 mm compensation block, making the attempt to compensate for a greater cartilage thickness technically demanding and potentially increasing the risk of errors.

An alternative approach would be to remove all cartilage from the unworn compartment, both distally and posteriorly, and apply a uniform 2 mm compensation for wear on both sides. This strategy, within the limitations of current instrumentation, would align the femoral component with the knee's kinematic axes relying directly on bone anatomy, with the compromise of a 1 mm anterior and proximal shift of the femoral component for every millimetre of prearthritic cartilage thickness exceeding 2 mm. Any resulting ‘excess’ in the extension and flexion space could then be easily compensated with a thicker polyethylene insert.

### Limitations

The major limitation of the current study is that both intraoperative methods relied on a millimetre ruler, allowing measurements only in 0.5 mm increments. Despite this, a 0.5 mm accuracy is considered acceptable in an operating room setting and aligns with the tolerated error in the calipered KA technique [21, 44]. To maintain consistency, decimal variations in cartilage thickness measurements obtained with the photographic method were also rounded to the nearest 0.5 mm, thus ensuring comparability among the three techniques.

Moreover, although after the bone cuts the bone–cartilage ‘biscuit’ was carefully split into two symmetrical halves and a scalpel was used to incise the cartilage perpendicularly to the cut surface, we acknowledge that a minimal degree of inaccuracy may have occurred. This inaccuracy could have introduced a measurement error due to the ‘parallax effect’. However, while this might have influenced results if measurements had been reported with decimal‐level precision, rounding to the nearest 0.5 mm effectively minimized its impact and rendered it negligible.

Finally, it could be argued that the two intraoperative methods were validated against cartilage measurements from photographed bone cuts, rather than preoperative Magnetic Resonance Imaging (MRI) images. While MRI is considered the gold standard of noninvasive cartilage assessment [3, 26, 43], its accuracy highly depends on several factors, including scan resolution [11, 50], proper image reconstruction in the kinematic sagittal plane [20], radiologist expertise and accurate definition of the bone–cartilage interface [51]. We believe that the photographic method can be reasonably considered as the ‘gold standard’ and the optimal reference method, as it is based on direct visualization of the cartilage layer [33] with a submillimetre accuracy in resolution. The validation of the described standardized intraoperative cartilage measurement techniques may allow a direct comparison with MRI measurements in future studies.

## CONCLUSION

Both intraoperative methods are reliable and comparable to the photographic method, with no significant differences among them. The electrocautery method has the added benefit of measuring cartilage thickness before any bone cuts are made, thereby avoiding potential bone recuts and/or compensatory adjustments.

## AUTHOR CONTRIBUTIONS

Giancarlo Giurazza had the idea for the article and was responsible for writing the manuscript. Stefano Campi and Edoardo Franceschetti were responsible for conceptualization and supervised data acquisition and analysis. Andrea Tanzilli was responsible for data analysis. Biagio Zampogna and Michele Paciotti were responsible for realization of figures and tables. Pietro Gregori qualified as the corresponding author. Michael T. Hirschmann and Rocco Papalia were responsible for reviewing and critically revise the manuscript. All authors have given final approval of the version to be published.

## CONFLICT OF INTEREST STATEMENT

The authors declare no conflicts of interest.

## ETHICS STATEMENT

The study was performed in accordance with the ethical standards as laid down in the 1964 Declaration of Helsinki and its later amendments. Institutional review board approval was obtained for this research (PAR 24.22 OSS). Written consent was obtained from all participants. No financial incentives were provided for participation.

## Data Availability

The data that support the findings of this study are available from the corresponding author [P. G.] upon reasonable request.
